# Exploring the Immediate and Long-Term Effects of Immersive Virtual Reality on Behavioral and Psychological Symptoms of Dementia and Caregiver Burden: Longitudinal Observational Study

**DOI:** 10.2196/73044

**Published:** 2025-07-16

**Authors:** Ling-Chun Huang, Ching-Fang Chien, Yuan-Han Yang

**Affiliations:** 1 Department of Neurology, Kaohsiung Medical University Gangshan Hospital, Kaohsiung Medical University, No. 8, Jie'an Road, Gangshan District, Kaohsiung, Taiwan; 2 Department of Neurology, Kaohsiung Medical University Hospital, Kaohsiung Medical University, Kaohsiung, Taiwan; 3 Neuroscience Research Center, Kaohsiung Medical University, Kaohsiung, Taiwan; 4 School of Post-Baccalaureate Medicine, College of Medicine, Kaohsiung Medical University, Kaohsiung, Taiwan; 5 School of Medicine, College of Medicine, Kaohsiung Medical University, Kaohsiung, Taiwan

**Keywords:** virtual reality, dementia, behavioral and psychological symptoms of dementia, caregiver burden, long-term effects

## Abstract

**Background:**

Virtual reality (VR) interventions are emerging as promising nonpharmacological strategies for people with dementia, aiming to prevent cognitive decline, reduce behavioral and psychological symptoms of dementia (BPSD), and alleviate caregiver burden. Although some studies have reported beneficial effects, findings remain inconsistent, and little is known about the duration and sustainability of these effects, particularly in real-world care settings.

**Objective:**

This study aimed to examine both the immediate and long-term effects of an immersive VR reminiscence intervention on BPSD and caregiver burden in people with dementia attending day care centers.

**Methods:**

This longitudinal observational study was conducted in 10 dementia day care centers in Kaohsiung, Taiwan. A total of 82 participants with dementia were enrolled. The VR intervention consisted of twice-weekly sessions over one month, featuring culturally familiar live-action 360° scenes filmed in well-known Taiwanese locations. Each session lasted approximately 10‐12 minutes and included interactive elements. Neuropsychiatric symptoms were assessed using the Neuropsychiatric Inventory Questionnaire, and caregiver burden was assessed using the Zarit Caregiver Burden Interview. Measurements were taken at 3 time points: preintervention, immediately postintervention, and 2 months after the intervention ended. The Wilcoxon signed-rank test was used for statistical comparisons, and rank-biserial correlation was calculated as the effect size.

**Results:**

Significant improvements were observed after 1 month of VR intervention in both caregiver burden (Z=−3.095, *P*=.002, *r*=0.34) and neuropsychiatric symptoms (Z=−2.929, *P*=.003, *r*=0.32). At the two-month follow-up, neuropsychiatric symptoms remained significantly improved (Z=−4.327, *P*<.001, *r*=0.48), although caregiver burden returned to preintervention levels. Regarding specific neuropsychiatric symptoms, significant improvements were observed immediately after the intervention in dysphoria or depression, anxiety, and sleep or nighttime behaviors. These effects were sustained over time, with additional long-term improvements noted in euphoria or elation, apathy or indifference, irritability or lability, aberrant motor behavior, and appetite or eating behaviors.

**Conclusions:**

A 1-month immersive VR reminiscence intervention appears to improve neuropsychiatric symptoms and temporarily reduce caregiver burden in people with dementia, with some symptom improvements lasting up to 2 months. These findings suggest that VR may offer a meaningful therapeutic option in day care settings. Future studies with control groups, including nonimmersive 2D conditions, and comparisons to traditional reminiscence therapy are needed to validate and expand upon these findings.

## Introduction

Over the past few decades, the rapid growth of the older adults population in high-income countries has led to a continuous rise in the prevalence of dementia, posing significant social and economic impacts [1]. Globally, more than 50 million people are estimated to have dementia, and this number continues to rise [2,3]. Behavioral and psychological symptoms of dementia (BPSD), also known as neuropsychiatric symptoms, encompass a wide range of noncognitive symptoms and behaviors, including agitation, aberrant motor behavior, anxiety, elation, irritability, depression, apathy, disinhibition, delusions, hallucinations, and changes in sleep or appetite. BPSD are frequently observed in patients with dementia, leading to distress, reduced quality of life, worsening cognitive and functional impairments, and increased caregiver stress and burden [4-6]. There is evidence that pharmacological treatments for BPSD often come with adverse side effects, including an increased risk of falls, fractures, stroke, and even mortality, while offering limited efficacy in symptom management [7]. Therefore, novel and effective nonpharmacological therapies with fewer side effects are urgently needed.

Virtual reality (VR) is a computer-generated environment that has been developed and refined in recent years. Using headsets, VR provides a fully immersive, highly engaging, and realistic experience. VR technologies are increasingly regarded as valuable tools in dementia research and as safe, well-tolerated nonpharmacological therapies that may enhance the quality of life and well-being of individuals with dementia [8-10]. Most studies investigating the impact of VR therapy on BPSD in people with dementia have been published since 2018 [10]. However, conclusions regarding its effectiveness have been somewhat inconsistent. For example, Rose et al [11] found that BPSD decreased during and after VR intervention compared to preintervention levels. In contrast, Coelho et al [12] reported no significant changes in the psychological and behavioral symptoms of participants following the intervention. Similarly, a scoping review examining empirical evidence on the use of fully immersive VR in reminiscence interventions found that some studies reported positive effects on anxiety, apathy, depression, cognitive function, and caregiver burden. However, these findings were inconsistent across other research [13]. Therefore, further investigation is required to better understand the impact of VR interventions on BPSD and caregiver burden.

Moreover, the long-term effects of VR interventions in people with dementia remain unclear [13]. In our previous study [14], we assessed cognitive function and depressive symptoms in 20 participants before and immediately after a VR intervention, with follow-up assessments conducted in 7 participants 3 to 6 months later. While cognitive abilities showed no significant changes immediately after the intervention, depressive symptoms were significantly reduced. However, cognitive function declined notably over the following months, suggesting that VR interventions may provide temporary cognitive benefits but not long-term preservation. It is important to note that the sample size was small, particularly for follow-up assessments, and only depressive symptoms of BPSD were evaluated in detail.

Therefore, we conducted this study to investigate both the immediate and long-term effects of immersive VR reminiscence therapy on BPSD and caregiver burden in people with dementia, with an increased sample size.

## Methods

### Study Design

This is a longitudinal observational study conducted in dementia day care centers to evaluate both the immediate and long-term effects of immersive VR reminiscence therapy on BPSD and caregiver burden. Participants were assessed before the intervention, immediately after the 1-month VR intervention, and 2 months postintervention.

### Recruitment

Participants were recruited from 10 dementia day care centers in Kaohsiung City, Taiwan. Staff at these centers identified potential participants based on predefined inclusion and exclusion criteria. The inclusion criteria were: (1) a diagnosis of all-cause dementia by experienced physicians, following the National Institute on Aging and Alzheimer’s Association diagnostic criteria [15]; and (2) regular attendance at the dementia day care centers between May 2023 and April 2024. Participants were excluded if they: (1) had difficulty recognizing VR images despite adjustments to the headset’s positioning; or (2) had cognitive impairment or BPSD severe enough to hinder proper assessment. Baseline demographic and functional information was collected using the Chinese version of the Uniform Data Set (UDS) Form A [16], which includes variables such as age, sex, years of education, marital status, living situation, and functional status (eg, need for assistance with complex or basic activities). The form was completed at enrollment by center staff, based on direct interviews with participants and input from their caregivers or informants.

### Ethical Considerations

The participants and their relatives were informed of the details of the study before their inclusion, and appropriate written informed consent was obtained. Given the vulnerability of individuals with dementia, additional precautions were taken to ensure comprehension, including clear verbal explanations and opportunities to ask questions. The Kaohsiung Medical University Hospital Institutional Review Board (KMUHIRB-SV(I)-20210015) approved the study protocol. All data were deidentified, stored securely, and accessible only to authorized researchers. Results are presented in aggregate to ensure confidentiality. Participants did not receive any financial compensation for their participation in this study.

### VR Apparatus

The HTC VIVE Pro VR head-mounted display (HMD; HTC Corporation) was used to deliver the immersive VR experience to the participants. It is a stand-alone HMD providing stereoscopic vision at a resolution of 2880×1600 per eye with a 90 Hz refresh rate. Two controllers were paired with the HMD to enable the participants to interact with the VR environment.

### Preparation of VR Content Implementation

**Figure 1.**
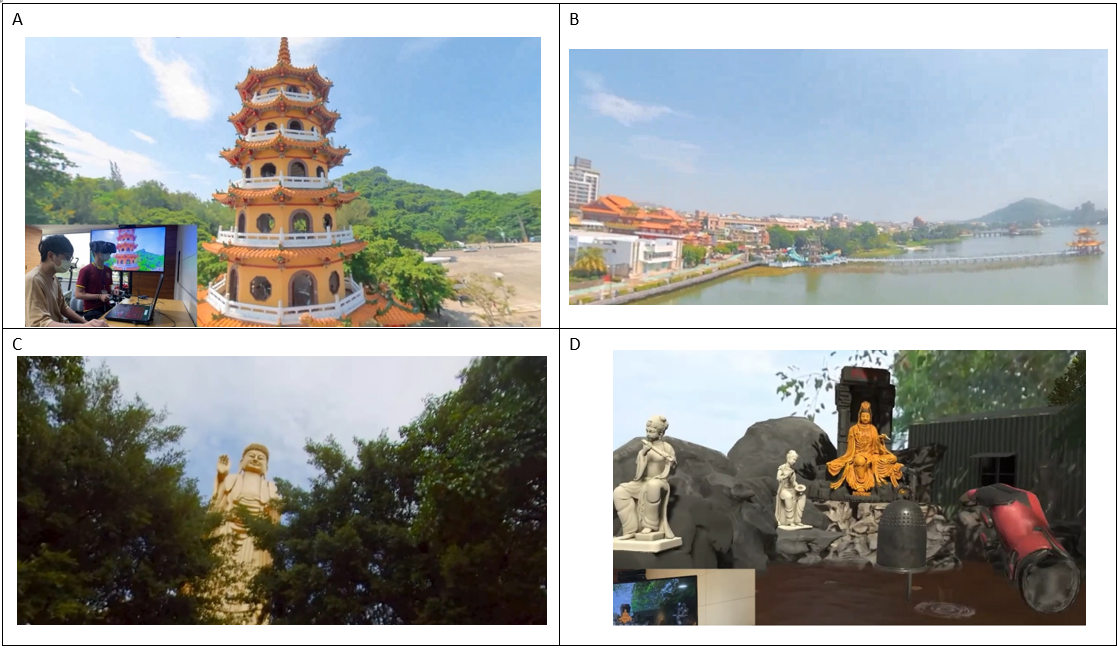
Scenes from the live-action virtual reality reminiscence intervention. The footage was captured at the (A,B) Lianchihtan (Lotus Pond) Scenic Area and the (C,D) Fo Guang Shan Buddha Museum in Kaohsiung City.

The VR content consisted of live-action scenes showcasing famous scenic areas and landmarks in Taiwan. These live-action images were captured using a 360° camera at Lianchihtan (Lotus Pond) Scenic Area and the Fo Guang Shan Buddha Museum in Kaohsiung City. Figure 1 displays screenshots from 2 of these scenes.

Lianchihtan (Lotus Pond) is one of Kaohsiung’s most historic landmarks and was listed as one of the “Eight Sights of Fongshan” during the Qing Dynasty. The Longhu Tower (Dragon Tiger Tower), renowned for its striking architectural design, is the most iconic attraction in Lianchihtan. Built in 1976, the seven-story tower features dragon and tiger sculptures as its symbolic entrance and exit. The walls are adorned with koji pottery created by various artists, depicting stories of religious and cultural significance. Visitors can take in a stunning 360-degree view of Lianchihtan’s scenic beauty, immersing themselves in its cultural and historical ambiance. The Buddha Museum fuses traditional and modern elements, offering facilities to assist in spiritual cultivation and the attainment of wisdom, while also serving as a hub for culture and education. The Lianchihtan Scenic Area and Fo Guang Shan Buddha Museum were selected for their cultural familiarity, high recognition, and potential to evoke personal memories in older Taiwanese adults. These well-known sites also offered rich sensory environments, which are beneficial for creating immersive and engaging VR experiences. To capture the essence of this remarkable site, we documented its various scenes through detailed photography. Digital reminiscence therapy recreates past scenes using real-time background removal technology, allowing users’ family members to be integrated into the VR environment. This creates a sense of togetherness despite physical distances. Users can be accompanied by family members or guided by a virtual tour guide while experiencing immersive elements like cicada sounds, drawing lots at the Great Buddha City, making wishes at the wishing pond, and tossing coins to ring a bell. In addition, the real-time background removal feature enables quick generation of shared moment photos with family.

### Implementation

The VR intervention was administered twice per week over a period of 1 month. The participants viewed and interacted with the VR content for approximately 10 to 12 minutes each time. The HMD images were mirrored to a laptop to enable the researcher to see what the participants were viewing and interacting with. The participants remained seated during the VR intervention to reduce motion sickness when using the HMD and to also minimize the risk of falls in the older adults. During each session, participants were guided through immersive VR scenes while the researcher encouraged reminiscence by prompting discussions about personal experiences related to the viewed locations. Open-ended questions such as “Have you visited this place before?” or “What does this remind you of?” were used to stimulate engagement. While the session structure remained consistent (10‐12 min, twice weekly), participants’ interactions varied based on their individual responses. The participants were provided with the controllers to enable them to interact with the VR space, and the researcher was beside them to assist and guide them when needed.

### Assessment of Questionnaire and Scores

Before, immediately after, and 2 months postintervention, the informants completed the Neuropsychiatric Inventory-Questionnaire (NPI-Q) to assess the severity of neuropsychiatric symptoms and their impact on them. Caregiver burden was evaluated using the Zarit Caregiver Burden Interview (ZBI).

#### Neuropsychiatric Inventory-Questionnaire

The NPI-Q was developed and cross-validated with the standard NPI to provide a brief assessment of neuropsychiatric symptomatology in patients with dementia for use in both clinical practice and research [17-20]. Adapted from the NPI [21], a validated informant-based interview assessing neuropsychiatric symptoms over the past month, the NPI-Q is designed as a self-administered questionnaire completed by caregivers. Each of the 12 NPI-Q domains contains a survey question that reflects cardinal symptoms of that domain. Initial responses to each domain question are “Yes” (present) or “No” (absent). If the response to the domain question is “No,” the informant goes to the next question. If “Yes,” the informant then rates both the severity of the symptoms present within the last month on a 3-point scale and the associated impact of the symptom manifestations on them (ie, caregiver distress) using a 5-point scale. The NPI-Q provides symptom severity and distress ratings for each symptom reported, and total severity and distress scores reflecting the sum of individual domain scores. An English version of the NPI-Q is provided in Multimedia Appendix 1.

#### Zarit Caregiver Burden Interview

The ZBI is a well-known self-reporting measure of perceived burden among caregivers [22], with prior applications in similar research on dementia [14,23]. The instrument measures the caregiver’s emotion, psychological health, well-being, social and family life, finances, and degree of control over one’s life. The version used contains 22 items, and each item on the questionnaire is a statement that the caregiver is asked to endorse on a 5-point Likert scale (0: never; 1: rarely; 2: sometimes; 3: quite frequently; and 4: nearly always) [22]. Higher scores indicate higher caregiver burden. An English version of the ZBI is provided in Multimedia Appendix 2.

### Statistical Analysis

In the results, the data are presented as mean (SD) or as a proportion. Comparisons of scores before and immediately after VR intervention, as well as before intervention and 2 months after stopping intervention, were performed using the Wilcoxon sign-rank test due to the nonnormal distribution of the dependent variables, as determined by the Kolmogorov-Smirnov test. For each Wilcoxon signed-rank test, the test statistic was converted into a standardized Z value, which reflects both the magnitude and direction of change. Effect sizes (*r*) were calculated using the rank-biserial correlation for each Wilcoxon signed-rank test. Based on Cohen convention [24], r value of approximately 0.1 indicates a small effect, approximately 0.3 a medium effect, and *r* ≥0.5 a large effect. To control for the false discovery rate resulting from multiple comparisons of the 12 individual NPI-Q domains, the Benjamini-Hochberg procedure was applied, with the false discovery rate set at 0.05. The required sample size was estimated using GPower 3.1 for a Wilcoxon signed-rank test. Assuming a large effect size (*r*=0.5), a significance level of 0.05, and a power of 0.80, the minimum required sample size was 64 participants. The large effect size was chosen based on Cohen guidelines due to the limited availability of prior data on immersive VR interventions for BPSD and caregiver burden. Analyses were performed using SPSS 26.0 (IBM Corporation). A 2-tailed *P* value of <.05 was considered to indicate a statistically significant difference.

## Results

### Baseline Demographic Characteristics

**Figure 2.**
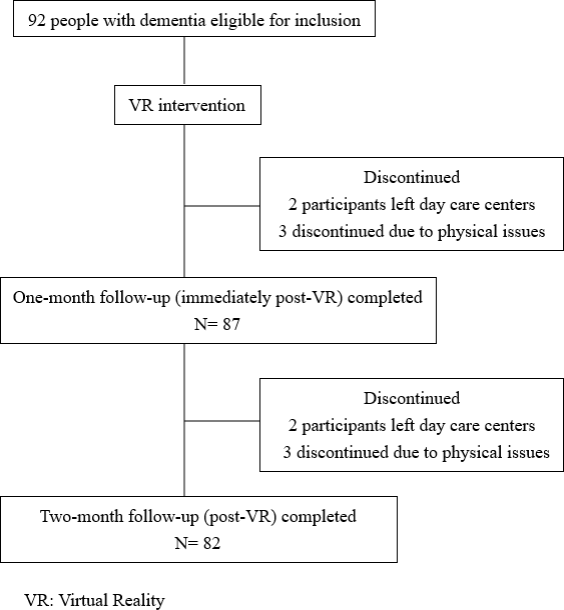
Diagram of the study design.

A total of 92 individuals were enrolled at the beginning of the study. After the 1-month intervention, 2 participants left the dementia day care centers, and 3 were unable to complete the postintervention evaluation due to physical issues. At the 2-month follow-up, an additional 2 participants left the centers, and 3 were unable to continue due to physical problems (Figure 2).

There were no statistically significant differences between the 2 groups (participants who completed the study versus those who dropped out) in terms of age, sex, education level, or baseline ZBI and NPI-Q scores. A detailed comparison between the 2 groups is presented in Table 1.

**Table 1.** Comparison of baseline characteristics between participants who completed the study and those who dropped out.

Variables	Nondrop out group (n=82)	Drop out group (n=10)	*P* value
Age in years, mean (SD)	79.7 (7.8)	81.5 (7.8)	.71
Sex, n (%)			.41
Male	27 (32.9)	2 (20)	
Female	55 (67.1)	8 (80)	
Education in years, mean (SD)	7.6 (3.1)	6.8 (1.5)	.21
Baseline ZBI^a^, mean (SD)	44.3 (18.0)	47.4 (17.8)	.46
Baseline NPI-Q^b^, mean (SD)	35.0 (23.1	29.7 (20.8)	.73

a ZBI: Zarit Caregiver Burden Interview.

b NPI-Q: Neuropsychiatric Inventory-Questionnaire.

The baseline demographic characteristics of the 82 participants who completed the study are shown in Table 2. The participants had a mean age of 79.7 years (SD 7.8), with 32.9% (27/82) being male and 67.1% (55/82) being female. The average education level was 7.6 years (SD 3.1). Most participants were categorized as requiring assistance with complex activities (35/82, 42.7%) or with basic activities (38/82, 46.3%). Regarding marital status, 73.2% (60/82) were widowed, and 20.7% (17/82) were married. For living situations, 81.7% (67/82) lived with a spouse, partner, or children; 7.3% (6/82) lived alone; and 7.3% (6/82) lived with a friend or other relative.

**Table 2.** Baseline demographic characteristics of the participants.

Variables	Participants (N=82)
Age in years, mean (SD)	79.7 (7.8)
Sex, n (%)	
Male	27 (32.9)
Female	55 (67.1)
Education in years, mean (SD)	7.6 (3.1)
Current marital status, n (%)	
Married	17 (20.7)
Widowed	60 (73.2)
Divorced	1 (1.2)
Separated	3 (3.7)
Never married	1 (1.2)
Level of independence, n (%)	
Independent	4 (4.9)
Assistance with complex activities	35 (42.7)
Assistance with basic activities	38 (46.3)
Completely dependent	5 (6.0)
Living situation, n (%)	
Alone	6 (7.3)
With spouse, partner, or children	67 (81.7)
With friend or other relative	6 (7.3)
Other	3 (3.7)

### The Immediate Effect of VR Intervention

Table 3 presents the immediate effect of VR intervention on neuropsychiatric symptoms and caregiver burden. Compared to preintervention levels, caregiver burden (ZBI) significantly decreased after the VR intervention (Z=−3.095, *P*=.002, *r*=0.34), and neuropsychiatric symptoms (NPI-Q) also showed a significant reduction (Z=−2.929, *P*=.003, *r*=0.32). Specifically, significant improvements were observed in dysphoria or depression (adjusted *P*=.036), anxiety (adjusted *P*=.036), and nighttime behaviors (adjusted *P*=.036).

**Table 3.** The immediate effect of virtual reality intervention on neuropsychiatric symptoms and caregiver burden.

Variables	Pre-VR^a^, mean (SD)	Immediately post-VR^a^, mean (SD)	*P* value^b^
ZBI^c^	44.3 (18.0)	38.0 (19.6)	.002^d^
NPI-Q^e^	35.0 (23.1)	28.6 (21.7)	.003^d^
Delusions	2.0 (4.0)	1.7 (3.4)	.34
Hallucinations	1.8 (3.9)	1.4 (3.3)	.31
Agitation or aggression	1.8 (3.7)	1.4 (3.1)	.48
Dysphoria or depression	3.3 (4.6)	2.0 (3.5)	.04^d^
Anxiety	3.2 (4.4)	2.0 (3.7)	.04^d^
Euphoria or elation	1.3 (3.4)	0.4 (1.3)	.08
Apathy or indifference	1.9 (3.5)	1.5 (2.9)	.61
Disinhibition	1.9 (3.9)	1.0 (3.0)	.10
Irritability or lability	2.3 (3.9)	1.3 (2.9)	.09
Aberrant motor	2.7 (4.5)	2.0 (3.7)	.08
Nighttime behavior	3.1 (5.2)	1.6 (3.3)	.04^d^
Appetite or eating	2.6 (4.9)	1.6 (3.4)	.10

a VR: Virtual Reality.

b *P* values comparing pre-VR and immediately post-VR using the Wilcoxon signed-rank test.

c ZBI: Zarit Caregiver Burden Interview.

d *P*<.05.

e NPI-Q: Neuropsychiatric Inventory-Questionnaire.

### The Long-Term Effect of VR Intervention

To assess the long-term effects of VR intervention, we conducted follow-up evaluations two months after the intervention ended. At the 2-month follow-up, caregiver burden remained unchanged (Z=−1.658, *P*=.097, *r*=0.18), while neuropsychiatric symptoms continued to improve (Z=−4.327, *P*<.001, *r*=0.48), indicating a moderate effect size. As shown in Table 4, significant improvements were observed in dysphoria and depression (adjusted *P*=.012), anxiety (adjusted *P*=.012), euphoria and elation (adjusted *P*=.008), apathy and indifference (adjusted *P*=.042), irritability and lability (adjusted *P*=.008), aberrant motor behavior (adjusted *P*=.015), sleep and nighttime behaviors (adjusted *P*=.008), and appetite and eating behaviors (adjusted *P*=.012).

**Table 4.** The long-term effect of virtual reality intervention on neuropsychiatric symptoms and caregiver burden.

Variables	Pre-VR^a^, mean (SD)	Two months post-VR^a^, mean (SD)	*P* value^b^
ZBI^c^	44.3 (18.0)	41.1 (19.5)	.10
NPI-Q^d^	35.0 (23.1)	25.7 (16.7)	<.001^e^
Delusions	2.0 (4.0)	1.4 (3.3)	.14
Hallucinations	1.8 (3.9)	1.2 (3.1)	.14
Agitation or aggression	1.8 (3.7)	1.0 (2.9)	.14
Dysphoria or depression	3.3 (4.6)	1.9 (3.2)	.01^e^
Anxiety	3.2 (4.4)	1.7 (3.2)	.01^e^
Euphoria or elation	1.3 (3.4)	0.2 (1.0)	.008^e^
Apathy or indifference	1.9 (3.5)	1.1 (3.0)	.04^e^
Disinhibition	1.9 (3.9)	0.9 (2.3)	.12
Irritability or lability	2.3 (3.9)	1.0 (2.3)	.008^e^
Aberrant motor	2.7 (4.5)	1.7 (3.7)	.02^e^
Nighttime behavior	3.1 (5.2)	1.5 (3.2)	.008^e^
Appetite or eating	2.6 (4.9)	1.1 (2.0)	.01^e^

a VR: Virtual Reality.

b *P*-values comparing pre-VR and two months post-VR using the Wilcoxon signed-rank test.

c ZBI: Zarit Caregiver Burden Interview.

d NPI-Q: Neuropsychiatric Inventory-Questionnaire.

e *P*<.05

## Discussion

### Principal Results

In this paper, we investigated the potential effects of immersive VR reminiscence therapy on people with dementia and their caregivers, as well as the duration of these effects. After 1 month of VR intervention, both caregiver burden and the severity of patients’ neuropsychiatric symptoms significantly decreased, with small to moderate effect sizes. In total, 2 months after the VR intervention ended, caregiver burden remained stable, while patients’ neuropsychiatric symptoms continued to improve with a moderate effect size, suggesting the sustained impact of VR therapy. VR was shown to reduce caregiver burden and improve neuropsychiatric symptoms, with symptom stability observed even 2 months postintervention. The improvements in euphoria, apathy, irritability, aberrant motor behavior, and appetite and eating behaviors may suggest delayed therapeutic effects. To the best of our knowledge, this is the first study to investigate not only the potential effects of immersive VR reminiscence on various neuropsychiatric symptoms but also the duration of these effects in individuals with dementia.

### Comparison With Previous Work

VR is increasingly recognized as a valuable therapeutic tool for people with dementia. It offers a cost-effective, noninvasive, and ethically acceptable approach to managing BPSD by redirecting patients’ attention within a physically safe environment [25]. Immersive VR therapy has shown potential in reducing aggressive behaviors among patients with dementia in acute care hospitals [26]. In addition, BPSD decreased during and after VR interventions in hospitalized individuals with dementia compared to preintervention levels [11]. Our study, conducted in a community care setting, similarly demonstrated a reduction in BPSD following VR intervention. However, a previous study promoting VR reminiscence therapy in individuals with dementia found no significant changes in psychological and behavioral symptoms or quality of life after a short intervention period [12]. Likewise, Mendez et al reported no significant differences in arousal, stress, anxiety, anger, fatigue, or attention between pre- and postintervention sessions [27]. These inconsistencies across studies may stem from small sample sizes, variations in dementia subtypes, differences in study designs, outcome measures, VR content, software delivery methods, and levels of interactivity. Despite these variations, VR-based reminiscence interventions generally foster high levels of interaction and immersion. Older adults, including those with mild cognitive impairment and dementia, often find these interventions enjoyable and engaging, suggesting potential therapeutic benefits.

Most studies focus on the immediate effects of VR interventions, but the duration of these effects remains unclear. We hypothesized that VR would lead to short-term symptom improvement immediately after the intervention and that some effects might persist over time. In our previous study [14], we assessed cognitive function and depressive symptoms in 20 participants before and immediately after VR intervention. In total, 7 participants were reassessed 3‐6 months later, revealing continued cognitive decline compared to postintervention levels. In this study, we examined the long-term effects of immersive VR intervention on BPSD and caregiver burden with a larger sample. Given that the NPI-Q assesses neuropsychiatric symptoms over the past month, we selected the time point of 2 months after the intervention ended to minimize measurement interference. Unlike cognitive function, neuropsychiatric symptoms remained improved compared to preintervention levels even 2 months after the intervention ended. This suggests that VR’s effects on neuropsychiatric symptoms may persist longer than its impact on cognitive function, lasting approximately twice the duration of the intervention. Further studies with longer follow-ups are needed to confirm this finding. Regarding specific neuropsychiatric symptoms, our results showed significant improvements in dysphoria or depression, anxiety, and sleep or nighttime behaviors immediately after the VR intervention. These improvements persisted over time, with additional long-term benefits observed in euphoria, apathy, irritability, aberrant motor behavior, and appetite and eating behaviors 2 months postintervention. These delayed improvements may reflect gradual engagement of motivational and emotional circuits, psychological adaptation processes, and evolving caregiver-patient dynamics, all of which may require time to unfold. In addition, limited but present neuroplasticity in older adults may contribute to slow-developing affective or behavioral changes [28]. These findings warrant further investigation to clarify the mechanisms driving delayed symptom changes.

Older adults with cognitive impairments may face challenges in accessing experiences beyond their physical surroundings. VR creates a 3D digital space that simulates real-world environments, allowing users to explore new places, revisit past memories, and engage meaningfully in life despite geographical or cognitive limitations [9]. In addition, networked VR allows multiple individuals to engage in shared experiences, such as group travel and coviewing virtual media, regardless of location. Its rich sensory stimuli create an immersive sense of presence, enabling older adults to feel truly connected with their loved ones [29]. In this study, although family members did not participate in the VR experience in real-time, their images were integrated into the virtual environment using real-time background removal technology. This allowed for interaction and created a sense of togetherness despite physical distance. Recent research has found that the use of VR to provide interactions may offer an alternative way of delivering stimulation to people with dementia who do not engage in other lifestyle activities [30]. In our study, participants were provided with controllers, enabling them to interact with the VR space (eg, drawing lots, tossing coins to ring a bell), which further enhanced their engagement in the scene and sparked their interest.

Our findings indicate that VR reminiscence intervention significantly reduced caregiver burden, consistent with the results of Yahara et al [31]. This reduction may be linked to the alleviation of neuropsychiatric symptoms, as these symptoms are strong predictors of increased caregiver burden over time. Longitudinal studies have shown that neuropsychiatric symptoms not only emerge early in dementia [32] but also tend to worsen as the disease progresses [33], significantly impacting caregivers. Among these symptoms, disruptive behaviors such as agitation or aggression, irritability, disinhibition, and aberrant motor behavior contribute the most to caregiver burden, followed by delusions and mood disturbances [34]. Dauphinot et al further found that apathy, agitation or aggression, aberrant motor behavior, appetite changes, and irritability were independently associated with caregiver burden, even after controlling for cognitive decline, functional impairment, and antidepressant use [35]. These findings suggest that VR interventions may help ease caregiver burden by mitigating neuropsychiatric symptoms. However, despite the sustained improvement in neuropsychiatric symptoms, caregiver burden remained unchanged 2 months after the intervention ended. This suggests that other factors may influence caregiver burden beyond symptom severity alone. Further research is needed to identify and address these additional contributing factors.

### Limitations

There were some limitations to this study. First, the absence of a control group, including a traditional reminiscence therapy group or a nonimmersive 2D control condition (eg, viewing similar content on a standard screen), requires careful interpretation of the results. Implementing such control conditions posed practical challenges. Assigning participants to wear a VR headset without content may cause discomfort; similarly assigning them to traditional reminiscence therapy or no intervention was deemed impractical due to the group-based nature of dementia day care centers. In addition, caregivers were supportive of the VR intervention, and excluding participants from the activity could have led to dissatisfaction. As a result, it is difficult to determine whether the observed effects were specifically attributable to the immersive nature of VR or to the reminiscence content itself. Moreover, natural symptom fluctuations, regression to the mean, or placebo effects could also have influenced the results. Despite this limitation, our study provides valuable insights into the real-world application of VR for dementia care. Future research should incorporate ethically appropriate control groups, including nonimmersive conditions, to strengthen causal inferences and further validate these findings. Second, potential participants were identified by the staff at dementia day care centers, which may limit the generalizability of our findings to the broader dementia population. In addition, individuals with severe cognitive impairments or BPSD were excluded, which is a common limitation in most VR studies, further restricting the applicability of the results. Third, during the follow-up period, 4 participants left the dementia day care centers, and 6 were unable to complete the follow-up evaluation due to physical problems. This represents a loss to follow-up rate of only 10.9%, which is below the 20% threshold, indicating a minimal risk of bias [36]. Fourth, this study did not include data on dementia severity and dementia subtype classification. However, in Taiwan, most dementia daycare centers recruit individuals with a clinical dementia rating of 1 or higher, with the majority being clinical dementia rating 1 (mild) and the remainder typically clinical dementia rating 2 (moderate). In clinical settings, more than half of dementia cases are attributed to Alzheimer’s disease. Furthermore, according to the study by Lora Appel et al [25], 70% of participants displayed enjoyment or relaxation during the VR session, with dementia severity ranging from mild (20%) to moderate (10%) and advanced (40%). It is worth noting that VR-based reminiscence interventions could be tailored to provide greater assistance for patients in the advanced stage, given their higher prevalence of BPSD. Fifth, caregiver burden in dementia is a complex and multidimensional construct that includes both patient and caregiver-related factors [37]. It has been associated with patients’ symptoms (such as BPSD, cognitive performance, and functional impairment), as well as personal characteristics. In this study, the ZBI was primarily completed by regular staff members at the dementia day care centers, as family caregivers were typically not present during assessment periods and often had limited availability. Therefore, the reported burden reflects the perspective of professional caregivers who interacted with the participants on a daily basis, rather than that of family members. Sixth, the observed effect sizes were smaller than the estimated large effect size, which may suggest that our study was underpowered to detect more modest effects. Future studies should consider using more conservative effect size assumptions when calculating sample size. Despite these limitations, to the best of our knowledge, this is the first study to explore not only the potential effects of immersive VR reminiscence but also the duration of these effects in people with dementia, using a relatively large sample size. Future studies could build on these results to develop personalized VR content tailored to different cognitive and emotional needs. Additionally, integrating VR into routine dementia care programs, such as day care centers or home-based interventions, may enhance accessibility and caregiver support. While more large-scale, controlled trials are needed to validate these effects, our study provides foundational evidence that could inform the design of future interventions and practical applications in dementia care.

### Conclusions

This study demonstrated that immersive VR reminiscence therapy has the potential to reduce caregiver burden and improve neuropsychiatric symptoms in people with dementia. Notably, the beneficial effects on patients’ symptoms persisted even two months postintervention. These findings suggest that VR reminiscence therapy may offer sustained and delayed therapeutic benefits, providing a promising nonpharmacological approach for dementia care.

## Supplementary material

10.2196/73044 Multimedia Appendix 1 English version of the Neuropsychiatric Inventory Questionnaire.

10.2196/73044 Multimedia Appendix 2 English version of the Zarit Caregiver Burden Interview.

## Abbreviations

BPSD: Behavioral and psychological symptoms of dementia
HMD: Head-mounted display
NPI-Q: Neuropsychiatric Inventory-Questionnaire
VR: Virtual reality
UDS: Uniform Data Set
ZBI: Zarit Caregiver Burden Interview
