# Supplementary material for: Exploring the Immediate and Long-Term Effects of Immersive Virtual Reality on Behavioral and Psychological Symptoms of Dementia and Caregiver Burden: Longitudinal Observational Study
Source: JMIR Serious Games. 2025 Jul 16;13:e73044. doi: 10.2196/73044 (PMC12286566; doi:10.2196/73044)
Supplement: Multimedia Appendix 1 [file games-v13-e73044-s001.pdf]

The Neuropsychiatric Inventory–Questionnaire

Please answer the following questions based on <u>changes</u> that have occurred since the patient first began to experience memory problems.

Circle "Yes" only if the symptom(s) has been present in the last month. Otherwise, circle "No". For each item marked "Yes":

a) Rate the SEVERITY of the symptom (how it affects the patient):

1 = Mild (noticeable, but not a significant change)
2 = Moderate (significant, but not a dramatic change)
3 = Severe (very marked or prominent, a dramatic change)

b) Rate the DISTRESS you experience due to that symptom (how it affects you):

0 = Not distressing at all
1 = Minimal (slightly distressing, not a problem to cope with)
2 = Mild (not very distressing, generally easy to cope with)
3 = Moderate (fairly distressing, not always easy to cope with)
4 = Severe (very distressing, difficult to cope with)
5 = Extreme or Very Severe (extremely distressing, unable to cope with)

	**No**	**Severity**	**Caregiver Distress**
**Delusions**	0	1 2 3	0 1 2 3 4 5
**Hallucinations**	0	1 2 3	0 1 2 3 4 5
**Agitation/Aggression**	0	1 2 3	0 1 2 3 4 5
**Dysphoria/Depression**	0	1 2 3	0 1 2 3 4 5
**Anxiety**	0	1 2 3	0 1 2 3 4 5
**Euphoria/Elation**	0	1 2 3	0 1 2 3 4 5
**Apathy/Indifference**	0	1 2 3	0 1 2 3 4 5
**Disinhibition**	0	1 2 3	0 1 2 3 4 5
**Irritability/Lability**	0	1 2 3	0 1 2 3 4 5
**Aberrant Motor**	0	1 2 3	0 1 2 3 4 5
**Nighttime Behavior**	0	1 2 3	0 1 2 3 4 5
**Appetite/Eating**	0	1 2 3	0 1 2 3 4 5
**TOTAL**			

Permission to use the instrument was granted for non-commercial academic purposes (© JL Cummings, 1994, all rights reserved).
