# Supplementary material for: Exploring the Immediate and Long-Term Effects of Immersive Virtual Reality on Behavioral and Psychological Symptoms of Dementia and Caregiver Burden: Longitudinal Observational Study
Source: JMIR Serious Games. 2025 Jul 16;13:e73044. doi: 10.2196/73044 (PMC12286566; doi:10.2196/73044)
Supplement: Multimedia Appendix 2 [file games-v13-e73044-s002.pdf]

Zarit Caregiver Burden Assessment

Name: ______________________________________ Date:____________________

The following is a list of statements that reflect how people sometimes feel when taking care of another person. After reading each statement, indicate how often you experience the feelings listed by circling the number that best corresponds to the frequency of these feelings.

	Never	Rarely	Sometimes	Frequently	Nearly Always
1) Do you feel stressed between caring for your relative and trying to meet other responsibilities for your family or work?	0	1	2	3	4
2) Do you feel embarrassed you're your relative's behavior?	0	1	2	3	4
3) Do you feel angry when you are around your relative?	0	1	2	3	4
4) Do you feel that your relative currently affects your relationship with other family members or friends in a negative way?	0	1	2	3	4
5) Are you afraid what the future holds for your relative?	0	1	2	3	4
6) Do you feel strained when you are around your relative?	0	1	2	3	4
7) Do you feel that you do not have as much privacy as you would like because of your relative?	0	1	2	3	4
8) Do you feel that your social life has suffered because you are caring for your relative?	0	1	2	3	4
9) Do you feel uncomfortable about having friends over because of your relative?	0	1	2	3	4
10) Do you feel that you have lost control of your life since your relative's illness?	0	1	2	3	4
11) Do you wish you could just leave the care of your relative to someone else?	0	1	2	3	4
12) Do you feel uncertain about what to do about your relative?	0	1	2	3	4

Over

	Never	Rarely	Sometimes	Frequently	Nearly Always
13) Do you feel that you should be doing more for your relative?	0	1	2	3	4
14) Do you feel you could do a better job in caring for your relative?	0	1	2	3	4
15) Overall, how burdened do you feel in caring for your relative?	0	1	2	3	4
16) Do you feel that your relative asks for more help than (s)he needs?	0	1	2	3	4
17) Do you feel that because of the time you spend with your relative that you do not have enough time for yourself?	0	1	2	3	4
18) Do you feel your relative is dependent upon you?	0	1	2	3	4
19) Do you feel your health has suffered because of your involvement with your relative?	0	1	2	3	4
19) Do you feel your health has suffered because of your involvement with your relative?	0	1	2	3	4
20) Do you feel that your relative seems to expect you to take care of him/her as if you were the only one he/she could depend on?	0	1	2	3	4
21) Do you feel that you will be unable to take care of your relative much longer?	0	1	2	3	4
22) Do you feel that you do not have enough money to care for your relative in addition to the rest of your expenses?	0	1	2	3	4

Scoring Instructions: Add Items 1-12 **Total 1-12 (maximum score = 48)** __________
Add Items 13-21 **Total 13-21 (maximum score = 36)** __________
Score #22 (maximum score = 4) ___________

Total Score (88) ________

Source: Zarit SH, Reever KE, Bach-Peterson J. Relatives of the impaired elderly: correlates of feelings of burden. The Gerontologist 1980; 20:649-655.
